# Circadian desynchronization disrupts physiological rhythms of prefrontal cortex pyramidal neurons in mice

**DOI:** 10.1038/s41598-023-35898-8

**Published:** 2023-06-06

**Authors:** Brandon L. Roberts, Ilia N. Karatsoreos

**Affiliations:** Neuroscience and Behavior Program, and Department of Psychological and Brain Sciences, University of Massachusetts Amherst, Tobin Hall, 135 Hicks Way, Amherst, MA 01003S USA

**Keywords:** Circadian mechanisms, Neuronal physiology

## Abstract

Disruption of circadian rhythms, such as shift work and jet lag, are associated with negative physiological and behavioral outcomes, including changes in affective state, learning and memory, and cognitive function. The prefrontal cortex (PFC) is heavily involved in all of these processes. Many PFC-associated behaviors are time-of-day dependent, and disruption of daily rhythms negatively impacts these behavioral outputs. Yet how disruption of daily rhythms impacts the fundamental function of PFC neurons, and the mechanism(s) by which this occurs, remains unknown. Using a mouse model, we demonstrate that the activity and action potential dynamics of prelimbic PFC neurons are regulated by time-of-day in a sex specific manner. Further, we show that postsynaptic K^+^ channels play a central role in physiological rhythms, suggesting an intrinsic gating mechanism mediating physiological activity. Finally, we demonstrate that environmental circadian desynchronization alters the intrinsic functioning of these neurons independent of time-of-day. These key discoveries demonstrate that daily rhythms contribute to the mechanisms underlying the essential physiology of PFC circuits and provide potential mechanisms by which circadian disruption may impact the fundamental properties of neurons.

## Introduction

Circadian (daily) rhythms are ubiquitous in nature and play a central role in health and disease. Synchrony between internal clocks and external time are important for optimal organismal function, while their desynchronization leads to myriad negative mental and physical health outcomes. Our previous work has shown that environmental circadian desynchronization, by exposing mice to altered day-night cycles, has profound impacts on behavioral flexibility and responses to novel environments^1^. However, the mechanisms remain elusive. Understanding the functional relevance of normal circadian rhythms in neural function is important if we are to fully appreciate how disrupted clocks impact health outcomes.

The prefrontal cortex (PFC) serves as a critical node in cognition, emotional systems, stress responses, and learning and memory, all of which vary over the course of the day^2–6^. It also displays diurnal changes in expression of the clock genes *Per1*, *Per2*, and *Bmal1*, in male and female rats^7,8^. The PFC is comprised of an array of cell types, including excitatory pyramidal neurons and inhibitory interneurons, which together impact behavior by relaying information to other brain regions including the amygdala and hippocampus, both of which also demonstrate circadian rhythms^9–12^. In mouse, the prelimbic area (pl) of the PFC is divided into six distinct layers, each with distinct inputs and projections. Specifically, neurons in layer 2/3 play a major role in working memory and behavioral plasticity and is involved in stress and depressive behaviors^10,13–15^. Remarkably, our previous work showed that the behavioral consequences of environmental circadian desynchronization (ECD) are associated with significant changes in the morphology of these layer 2/3 plPFC pyramidal neurons^1^. Disruption of the light-cycle by reversal of the light–dark period every three days, also changes the expression of numerous clock genes within the PFC^16^. Yet the impacts of circadian desynchronization on neuronal function remain unknown.

The majority of PFC pyramidal neurons are intrinsically quiescent in their resting state and regulate information throughput via a wide array of ion channels, such as cyclic-nucleotide-gated non-selective cation (HCN) channels, sodium (Na^+^), calcium (Ca^2+^) and potassium (K^+^) channels, including g-protein inward rectifying K^+^ (GIRK) channels that mediate postsynaptic throughput of synaptic currents^14,16–18^. In the suprachiasmatic nucleus (SCN) brain clock, changes in sodium (Na^+^), K^+^, and Ca^2+^ ion channel function mediate daily rhythms in the spontaneous activity, and action potential dynamics of neurons^19,20^. How these channels might impact time-of-day changes in PFC function and neural excitability remains an important gap in our knowledge.

In the present set of studies, we demonstrate that time-of-day clearly drives changes in the fundamental neurophysiological properties of these neurons, including activity and excitability, defined here as a combination of resting membrane potential, action potential threshold, and action potential firing rate after current injection. Second, we identify that a potential mechanism for these daily changes in the plPFC involves rhythms in K^+^ channel function. Finally, we show that circadian desynchronization markedly alters the intrinsic function of plPFC pyramidal neurons. Our findings reveal that time-of-day is a critical factor that significantly changes neurophysiological properties of plPFC pyramidal neurons and that environmental circadian desynchronization has sizeable consequences for neural function independent of time-of-day. Together, this work further establishes these cells as an important substrate for some of the negative outcomes that accompany circadian desynchronization.

## Results

### Resting membrane potential of prelimbic layer 2/3 pyramidal neurons is rhythmic in male mice

The regional and cell-specific heterogeneity in electrophysiological properties of PFC pyramidal neurons has been described in multiple species^14,21,22^. Here we define type I pyramidal neurons as those that are quiescent (non-firing) at rest, slow firing after stimulation, have a longer half-width, and shallow afterhyperpolarization (AHP), while all other phenotypes are considered unidentified type II neurons. These characterizations are in line with in vivo work using spike sorting techniques to distinguish between glutamatergic pyramidal neurons and GABAergic interneurons^23^, some of which can morphologically appear pyramidal-like in shape.

**Figure 1:**
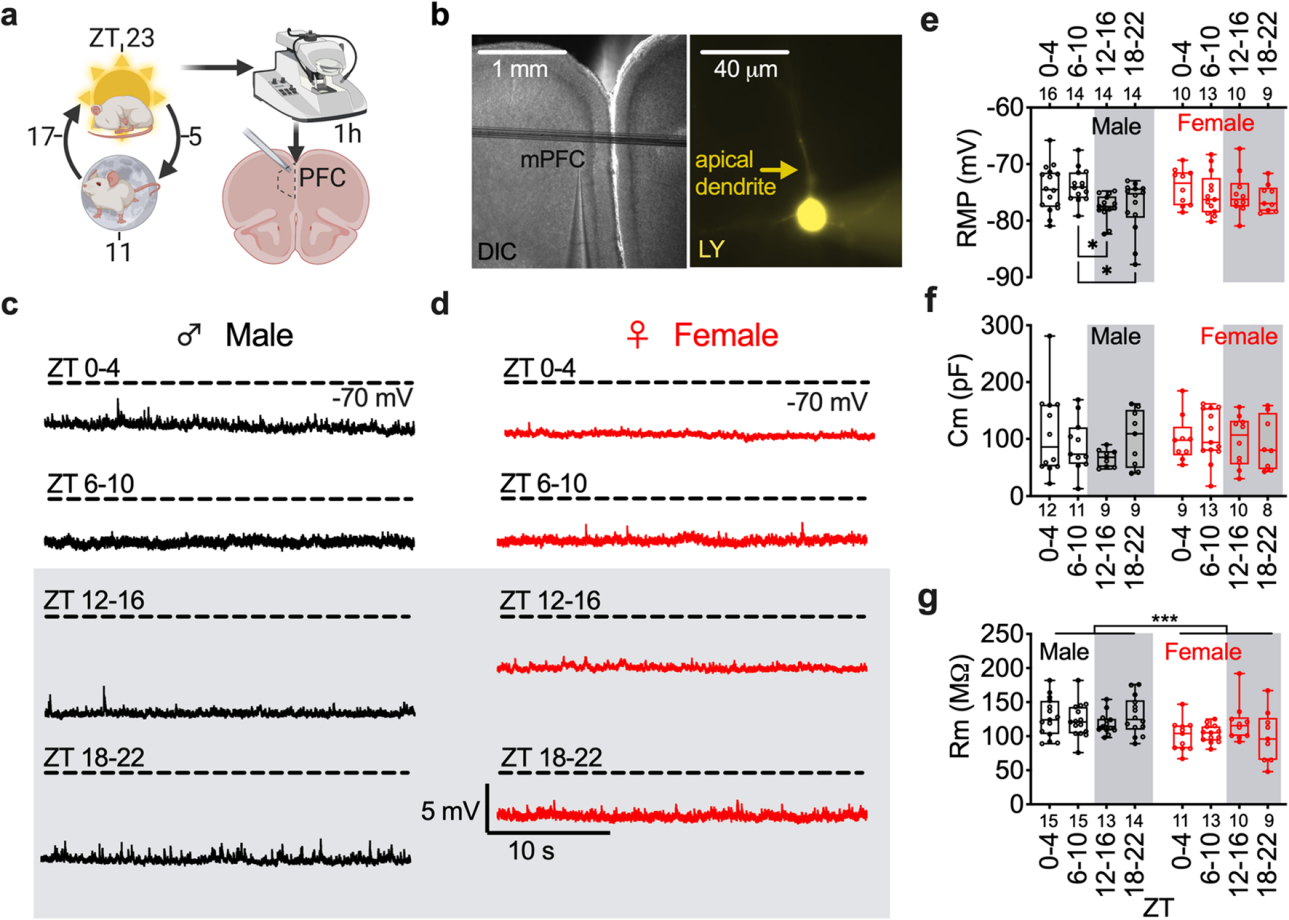
Resting membrane potential of layer 2/3 mPFC pyramidal neurons is rhythmic in male mice. (**a**) Diagram of methodological approach for ZT times (left) of ex vivo slice collection (right) (**b**) mPFC slice (left*;* scale 1 mm) and layer 2/3 pyramidal neuron backfilled with lucifer yellow (LY, right*;* scale 40 µm). (**c**) Representative traces of current clamp recordings from male and (**d**) female mice at each ZT bin. (**E**) Boxplot with individual data points for membrane potential (RMP) at ZT0–4, 6–10, 12–16, and 18–22 in male (black; left) and female (red; right) mice (Time: F (3, 92) = 3.126; *p* = 0.03, sex: F (1, 92) = 0.718; *p* = 0.40) (**f**) membrane capacitance (Cm; Time: F (3, 73) = 0.765, *p* = 0.52, sex: F (1, 73) = 0.430) and (**g**) resistance (Rm; Time: F (3, 92) = 0.250; *p* = 0.86, Sex: F (1, 92) = 13.25; *p* ≤ 0.001) binned by ZT. Box plots represent median, min/max, and second and third quartiles**.** N-values for number of cells inset on x-axis. Two-way ANOVA for main effects and interaction with a within group Tukey post-hoc analysis for ZT bin, *p < 0.05; ***p < 0.001.

We focused the present studies on the type I pyramidal subset, which composed ~ 80% of recorded neurons (Supplemental Information, Fig. S2A–H). The type I subset of layer 2/3 pyramidal neurons of the plPFC were identified visually by anatomical location and phenotypically by electrophysiological characterization in ex vivo coronal brain slices (Fig. 1a,b; Supplemental Information, Fig. S1A–D, Fig. S2A–H). Pyramidal neurons were identified by shape and lucifer yellow (LY; 0.2%) was added to the patch pipette for confirmation of an apical dendrite (Fig. 1b; right).

In pursuit of the mechanistic underpinnings for how ECD impacts the physiology of plPFC pyramidal neurons, we first needed to define how time-of-day impacts the normal function of these neurons. To test our hypothesis that time-of-day alters the basal electrophysiological properties of pyramidal neurons, we used whole-cell patch clamp techniques and measured resting membrane potential (RMP), capacitance (Cm) and membrane resistance (Rm) at Zeitgeber time (ZT) bins: 0–4, 6–10, 12–16, and 18–22 in male (*n* = 6 mice/group) and female mice (*n* = 4–6 mice/group) under a standard 12:12 light dark cycle (Fig. 1a–g). There was a main effect of time on RMP (no effect of sex) and notably, within group post-hoc analysis revealed that plPFC pyramidal neurons are more depolarized at ZT6-10 (light period) in male mice, when compared to 12–16 and 18–22 (dark period). Post-hoc analysis did not reveal a time-of-day effect on RMP in female mice (Fig. 1e). We did not observe a main effect of time on Cm or Rm; however, there was a main effect of sex on Rm (Fig. 1f,g). Together, these data demonstrate that the RMP of plPFC pyramidal neurons in male mice changes throughout the light/dark (LD) cycle.

### Action potential threshold is decreased during the active period in plPFC pyramidal neurons

**Figure 2:**
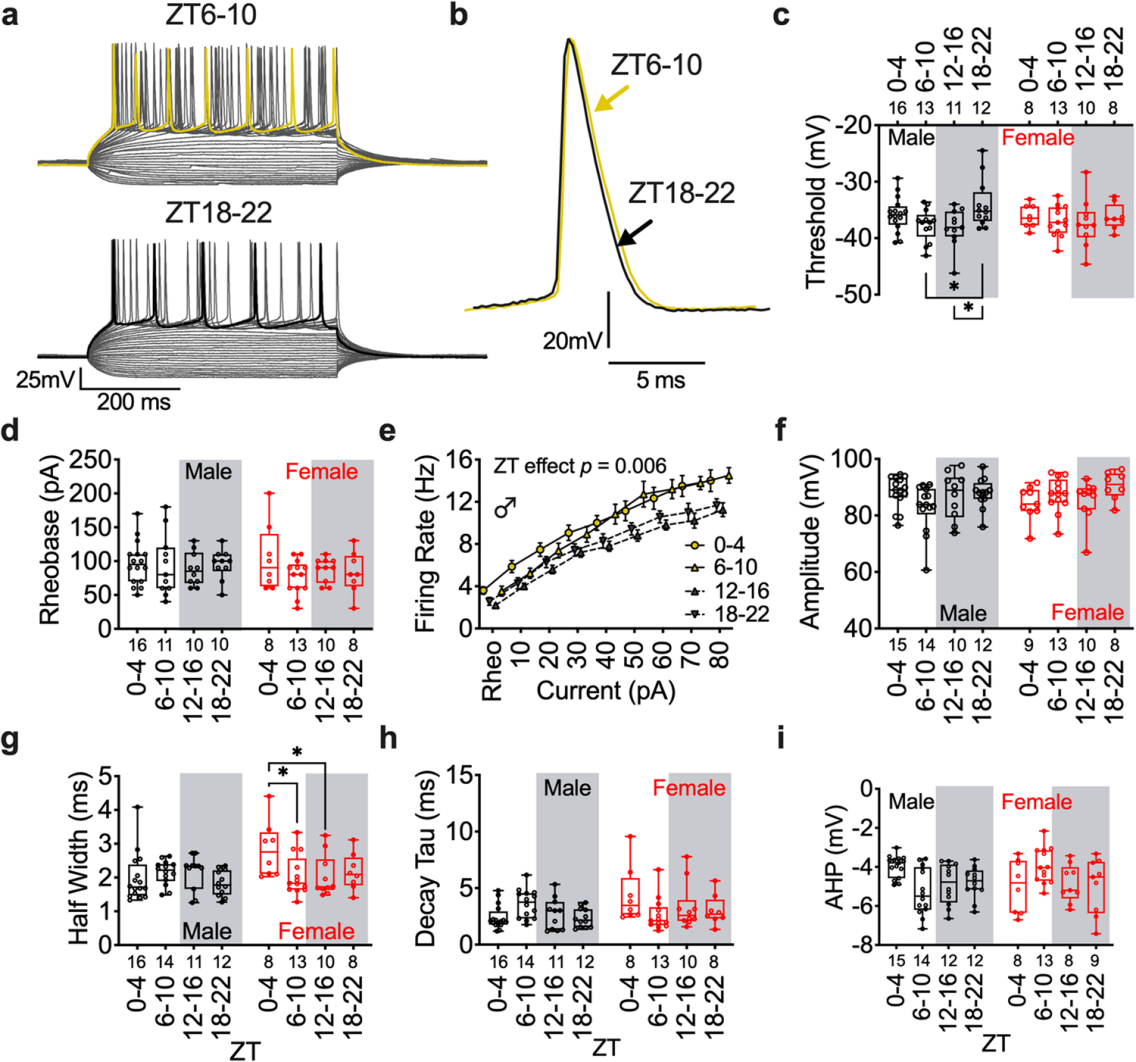
Neural excitability is decreased during the active period in male mice. (**a**) Representative trace of current step recording with maximal action potential (AP) firing highlighted at ZT6-10 (top; yellow) and 18–22 (bottom; black) and (**b**) individual evoked APs in male mice. (**c**) Boxplot and individual datapoints for AP threshold (Time: F (3, 83) = 2.809; *p* = 0.04, Sex: F (1, 83) = 0.1388; *p* = 0.71) (**d**) rheobase (Time: F (3, 78) = 0.9519; *p* = 0.42, Sex: F (1, 78) = 0.6786; *p* = 0.41), (**e**) evoked firing rate (from rheobase; male mice Time: F (3, 41) = 4.815; *p* = 0.006), (**f**) amplitude (Time: F (3, 83) = 1.489; *p* = 0.22, Sex: F (1, 83) = 0.1132; *p* = 0.74), (**g**) half-width (F (3, 84) = 1.993; *p* = 0.12, Sex: F (1, 84) = 4.045; *p* = 0.049), (**h**) decay tau (Time: F (3, 84) = 0.7564; *p* = 0.5217, Sex: F (1, 84) = 3.442; *p* = 0.0671) and (**i**) afterhyperpolarization (AHP) (Time: F (3, 84) = 0.8137; *p* = 0.49, Sex: F (1, 84) = 0.0505; *p* = 0.82) at each ZT bin in male and female mice. Box plots represent median, min/max, and second and third quartiles. Error bars on firing rate plots represent ± SEM. N-values for number of cells inset on x-axis. Two-way ANOVA for main effects and interaction, with a within group Tukey post-hoc analysis for ZT bin and/or current injection. *p < *0.05.*

To understand the functional implications of daily rhythms in RMP and postsynaptic ion channel function for neural excitability, we tested how time-of-day impacts action potential dynamics. We utilized a 10-pA current injection protocol to evoke action potentials at ZT0–4, 6–10, 12–16, and 18–22 and observed a main effect of time for membrane potential threshold of action potential firing, with post-hoc analysis revealing an increased threshold for firing late in the dark period (ZT18-22) when compared to ZT6-10 and 12–16 in male mice (*n* = 6 mice/group) (Fig. 2a–c). Consistent with the null effect of time on RMP in female mice, there was no effect of time on action potential threshold (*n* = 4–6 mice/group) (Fig. 2c). Further, although there was no effect on amount of current needed to elicit an action potential (rheobase) in male mice, once rheobase was reached, subsequent current injections evoked action potential firing at a lower frequency during the dark period (Fig. 2d,e). We did not observe a time-of-day effect on most other action potential dynamics in male mice, such as amplitude, half-width, decay tau, or afterhyperpolarization (AHP) (Fig. 2f–i). Together, these data suggest that plPFC pyramidal neurons are not only more hyperpolarized during the light period, but are functionally more difficult to activate, requiring larger depolarizations to elicit action potentials and relay information downstream.

### Membrane conductance is increased prior to the inactive period in female mice

**Figure 3:**
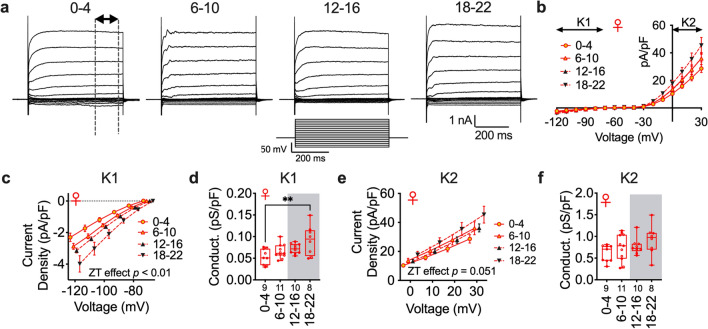
Membrane conductance at hyperpolarized voltages is increased during the active period in female mice.(**a**) Current–voltage trace (top; dashed line represents steady state current averaged for analysis) after voltage-step protocol (bottom middle). (**b**) Averaged I–V voltage-step relationship from − 120 to 30 mV for each ZT bin (normalized to cell capacitance) with a K^+^ internal solution. (**c**,**e**) Current density (K1 (time): F (3, 34) = 5.425; *p* = 0.0037, K2 (time): F (3, 34) = 2.869; *p* = 0.0508) and (**d**,**f**) conductance of K1 (F (3, 34) = 4.900; *p* = 0.006) and K2 (F (3, 34) = 1.938; *p* = 0.14) currents (respectively) at ZT0–4, 6–10, 12–16, and 18–22 in female mice. K1 and K2 represent hyperpolarized and depolarized currents (respectively). Box plots represent median, min/max, and second and third quartiles. Error bars on I–V plots represent ± SEM. N-values for number of cells inset on bars. Two-way (**c**,**e**) or One-way (**d**,**f**) ANOVA with Tukey post-hoc analysis for ZT bin. **p < 0.01.

To identify any potential daily changes in cell endogenous properties of pyramidal neurons in female mice, we next investigated how time-of-day impacts the cellular current–voltage (I–V) relationship. Changes in ion channel properties can be assessed by changes in current density and cellular conductance as measured by the I–V relationship. We used a potassium (K^+^) gluconate internal solution and performed an inactivation protocol in which neurons held at − 70 mV were stepped from 30 to − 120 mV, with subsequent − 10 mV sweeps. At each ZT bin, we analyzed the steady-state current density (current normalized to cell capacitance) of the inward (− 120 to − 70 mV steps (*K1*); Fig. 3a–c) and outward current (0–30 mV steps (*K2*); Fig. 3a,b,e), as well as daily changes in *K1* and *K2* conductance (*g*) normalized to cell capacitance (Fig. 3d,f). We observed a main time-of-day effect (*n* = 4–6 female mice/group) on current density and normalized conductance for the K1 inward current (Fig. 3c,d), although there was no main effect on the K2 outward current (Fig. 3b,e,f). Together, these data demonstrate that female mice do indeed display daily changes in postsynaptic membrane properties, but they are insufficient to alter resting state (Fig. 1d,e).

### K^+^ channel activity regulates postsynaptic rhythmic activity in pyramidal neurons of male mice

**Figure 4:**
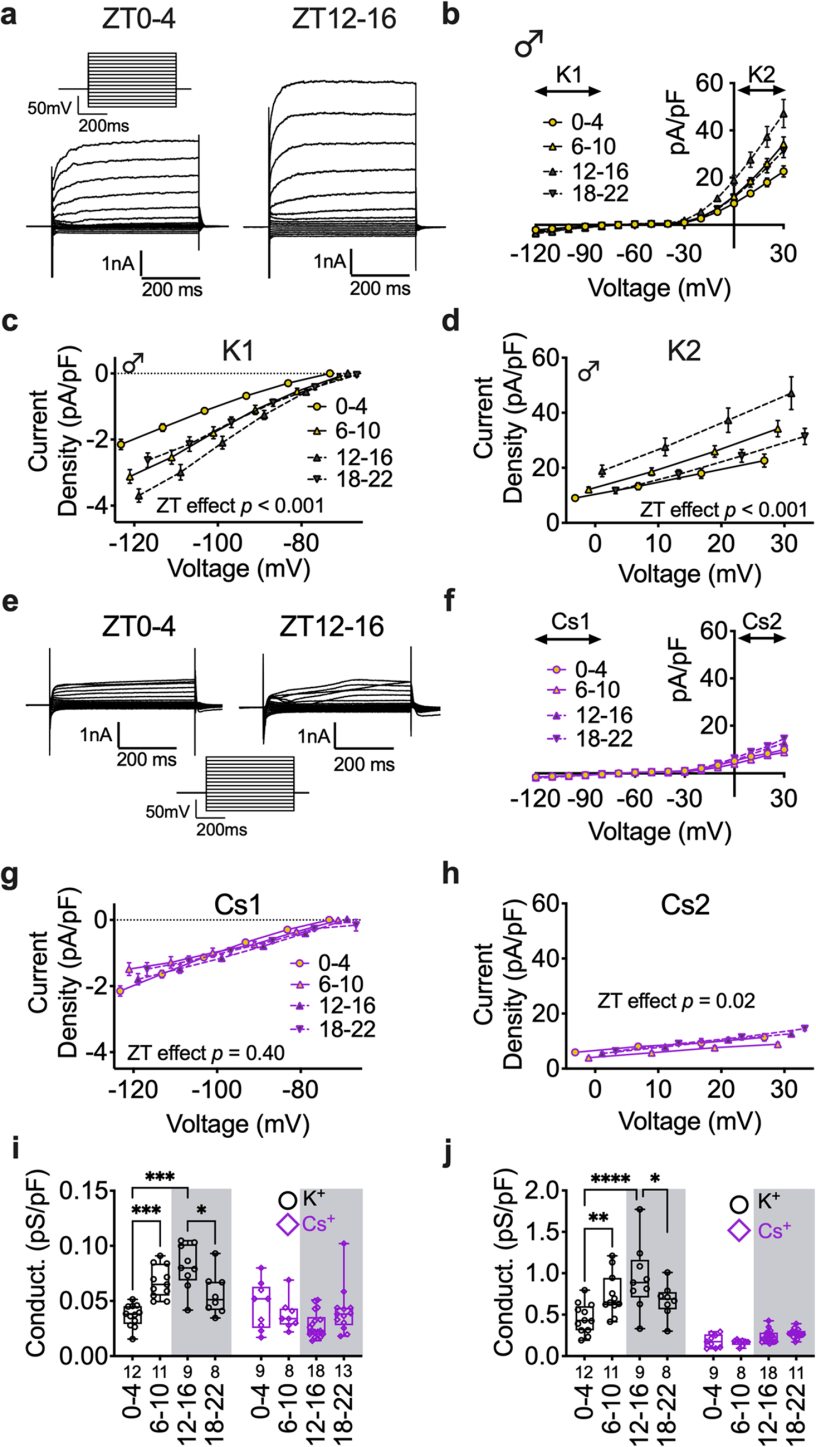
K^+^ channels are required for time-of-day effects on membrane conductance.(**a**) Voltage-step protocol (top left) and representative voltage-step traces of I–V relationship at ZT 0–4 (left) and 12–16 (right) in male mice. (**b**) Averaged I–V relationship for each ZT bin (normalized to cell capacitance) with a K^+^ internal solution. (**c**) Current density of K1 (Time: F (3, 37) = 9.115; *p* ≤ 0.001) and (**d**) K2 (Time: F (3, 37) = 9.055; *p* ≤ 0.001) I–V relationships at ZT0–4, 6–10, 12–16, and 18–22. (**e**) Representative voltage-step traces of (**f**) I–V relationship at ZT 0–4 (left) and 12–16 (right) with a Cs + internal solution. (**g**) Current density of Cs1 (Time: F (3, 48) = 1.009; *p* = 0.40) and (**h**) Cs2 (Time: F (3, 45) = 3.540; *p* = 0.02) I–V relationships at each ZT bin. (**i**) Comparison each ZT bin for K1 and Cs1 (Time: F (3, 80) = 2.682; *p* = 0.05, Internal: F (1, 80) = 37.22; *p* ≤ 0.001), and (**j**) K2 and Cs2 (Time: F (3, 81) = 7.896; *p* = 0.0001, Internal: F (1, 81) = 141.1; *p* ≤ 0.0001) normalized cell conductance. Two-way ANOVA for main effects and interaction, with a within group Tukey post-hoc analysis for ZT bin, voltage, and internal solution. Box plots represent median, min/max, and second and third quartiles. Error bars on I–V plots represent ± SEM. N-values for number of cells inset on x-axis. *p < 0.05, **p < 0.01, ***p < 0.001, ****p < 0.0001.

Given that female mice display daily changes in membrane conductance, but only male mice display daily rhythms in RMP, we hypothesized that changes in ion channel properties may play a role in setting the functional tone of plPFC pyramidal neurons in male mice. Similar to above, at each ZT bin (*n* = 6 male mice/group) we analyzed the steady-state current density of the inward (*K1*; Fig. 4a–c) and outward current (*K2*; Fig. 4a,b,d), as well as daily changes in *K1* and *K2* conductance normalized to cell capacitance (Fig. 4i,j). When compared among all ZT time bins, the I–V relationship demonstrated a clear increase in inward current at hyperpolarized holding voltages and an increase in delayed outward rectifying current at depolarized voltages early in the dark period (ZT12–16) (Fig. 4a–d). This effect translated into increased cell conductance late in the light period and early in the dark period, or near the transition to lights off (Fig. 4i,j).

Since ionic conductance was highest at ZT12–16, the same ZT bin that RMP was most hyperpolarized (Fig. 2b), we predicted that this increased conductance was due to increased K^+^ channel activity. To determine if daily changes in current density and cell conductance was dependent on K^+^ channel activity, we utilized a K^+^ free Cs^+^-based internal recording solution to block K^+^ currents (*n* = 6–8 male mice/group). This preparation completely abolished the time-of-day effect on inward currents and greatly reduced the time-of-day effect on outward currents (Fig. 4e–h). Further, the time-of-day effect on cell conductance was blocked by Cs^+^ (Fig. 4i,j). Of particular note, blockade of inward K^+^ currents via internal Cs^+^ appeared to have little effect at ZT0–4, suggesting minimal K^+^ channel conductance at this ZT bin (Fig. 4c,g). Together, these data demonstrate that K^+^ channels contribute to daily rhythms in the cellular conductance of plPFC pyramidal neurons.

### Circadian desynchronization decreases excitability of PFC pyramidal neurons

**Figure 5:**
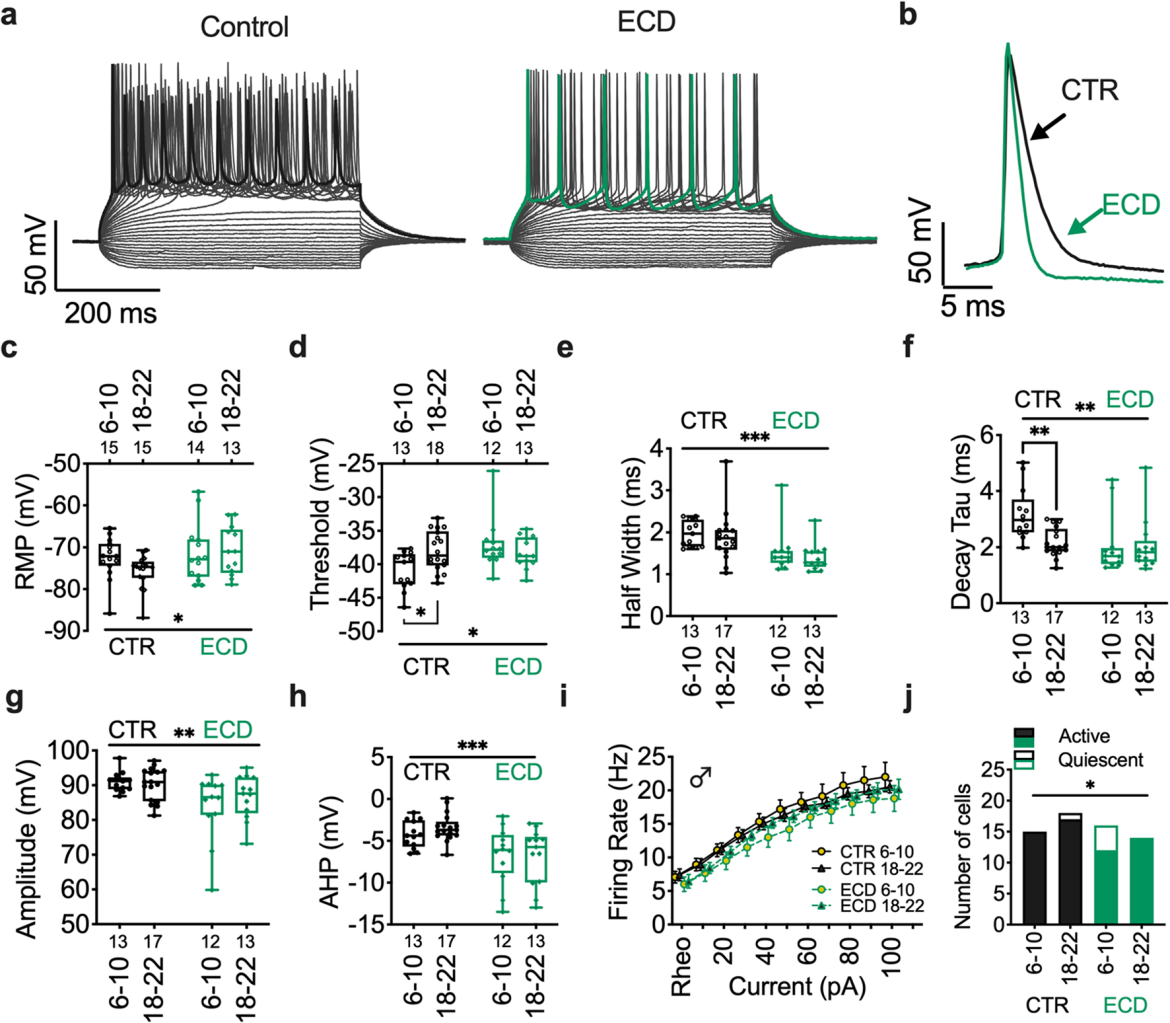
Environmental circadian desynchronization alters fundamental properties of PFC pyramidal neurons independent of time-of-day. (**a**) Representative trace of current step recording with maximal AP firing and (**b**) individual evoked APs highlighted at ZT6-10 in control (CTR) (left; black) and ECD (right; bluish green) in male mice. (**c**) Boxplot and individual datapoints showing membrane potential (Time: F (1, 52) = 0.9617; *p* = 0.33, Treatment: F (1, 52) = 4.275, *p* = 0.04), (**d**) action potential firing threshold (Time: F (1, 52) = 1.1143; *p* = 0.29, Treatment: F (1, 52) = 4.867; *p* = 0.03), (**e**) half width (Time: F (1, 51) = 0.8106; *p* = 0.37, Treatment: F (1, 51) = 13.80; *p* < 0.001), (**f**) decay tau (Time: F (1, 51) = 4.705; *p* = 0.0348, Treatment: F (1, 51) = 7.294; *p* = 0.0094) , (**g**) amplitude (Time: F (1, 51) = 0.4232; *p* = 0.52, Treatment: F (1, 51) = 10.24; *p* = 0.002), and (**h**) afterhyperpolarization (AHP; Time: F (1, 51) = 0.292; *p* = 0.5913, Treatment: F (1, 51) = 17.70; *p* = 0.0001) at ZT 6–10 and 18–22 in male mice. (**i**) Firing rate (F (3, 54) = 0.832; *p* = 0.48), Treatment: F (1, 53) = 1.081; *p* = 0.30) and (**j**) number of active versus quiescent neurons after 200pA current injection. Box plots represent median, min/max, and second and third quartiles. N-values for number of cells inset on x-axis. Two-way ANOVA for main effects and interaction, with Šídák's multiple comparisons post-hoc analysis for ZT bin. *p < 0.05; **p < 0.01; ***p < 0.001.

Environmental circadian desynchronization (ECD), implemented by housing in a 10:10 h light:dark cycle, negatively impacts cognitive flexibility and alters both morphology and dendritic connections of layer 2/3 plPFC pyramidal neurons in mice^1^. We hypothesized that the impact of ECD on these neurons would extend to their endogenous physiological properties. To test this, we used whole-cell patch clamp techniques and measured changes in resting state properties and action potential dynamics at ZT 6–10 and 18–22 (calculated as 50 min/h in ECD mice; Fig. 5a–h). We observed a main effect of ECD, independent of time-of-day, on the resting membrane potential and action potential firing threshold (Fig. 5c,d). Further, ECD had a main effect on all components of action potential dynamics that were measured, including half width, decay tau, amplitude, and AHP (Fig. 5e–h). Finally, all other effects of time observed in control mice, such as action potential threshold and decay tau, was lost in ECD mice. While there was no ECD effect on evoked action potential firing rate (as calculated from rheobase) after current injection (Fig. 5i), we did not include neurons that failed to elicit action potentials after current injections. In a separate Chi-squared analysis of active versus quiescent neurons, we observed a significant increase in the number of quiescent neurons in ECD mice (Fig. 5j). Together these data show that independent of time-of-day, ECD alters the fundamental intrinsic properties of plPFC pyramidal neurons.

## Discussion

Given the widespread realization that time of day significantly impacts behavioral function, and that disruption of the circadian clock leads to significant negative behavioral outcomes, understanding the impacts of biological time on neural circuit function is critical. While there have been several well executed studies that demonstrate the presence of daily rhythms in neurophysiological function in the hippocampus and brainstem, none have included the PFC, and many have largely focused on extracellular field recordings exclusively in males^23–27^. Thus, previous studies have not shed light on how circadian desynchronization impacts the fundamental electrophysiological processes in higher brain areas at a cellular level. In this study we present four main findings. First, we demonstrate that in male mice, layer 2/3 plPFC pyramidal neurons are hyperpolarized during the early portion of the dark period when compared to the latter portion of the light period. Second, we demonstrate that male mice display distinct changes in excitability, showing increased action potential firing threshold and decreased firing rate during the dark period. Third, we identify that changes in K^+^ channel activity may serve as a potential mechanism underlying time-of-day changes in the RMP and action potential firing rates of plPFC pyramidal neurons. Finally, we illustrate that circadian desynchronization disrupts multiple properties of neural function and reduces excitability of layer 2/3 plPFC pyramidal neurons independent of time-of-day. By identifying the intrinsic properties of plPFC pyramidal neurons with a putative cellular substrate, and how circadian desynchronization impacts the function of PFC neurons, these findings allow us to better understand the relationship between time-of-day, intrinsic PFC circuitry and its outputs, and sets the stage for future work to explore how endogenous circadian clocks might impact neural function.

Changes in PFC function underly numerous psychiatric disorders including bipolar, post-traumatic stress disorder (PTSD), attention deficit disorder, and deficits in learning and memory^4–6,28^. There is growing evidence of links between circadian rhythms and both PFC function and many of these behavioral conditions^2,29–32^. Previous work from our group has demonstrated that extracellular lactate (a functional output of neural metabolism) shows circadian rhythms in the medial (m) PFC (even in constant darkness), and that ECD alters the morphology of mPFC pyramidal neurons and PFC mediated behaviors^1,33^. The present studies contextualize these results and are the first to directly test whether cell autonomous activity of PFC neurons are modulated by time-of-day, define contributing mechanisms by which this occurs, and identify how environmental circadian disruption impacts these functions.

Our finding that the resting state of plPFC pyramidal neurons is more hyperpolarized during the dark period, when nocturnal mice are awake and active, suggests that these neurons are more quiescent and require a higher degree of information input (i.e. synaptic activation) before eliciting a response and sending downstream signals to other brain regions. That is, there is increased gating in this circuit at night. On the surface, it seems counter-intuitive that plPFC pyramidal neurons would show more gating during the dark (active) phase, than the light (rest) phase. A functional hypothesis for this finding is that stronger gating during the active phase serves as a mechanism for selective information throughput in response to environmental stimuli. Information filtering is paramount for proper behavioral output, and too low of a threshold may result in overactivation as the animal engages with its environment. For example, pharmacological studies have demonstrated that activation of the plPFC with neurotensin agonists or the sodium channel activator veratrine lead to anxiogenic behaviors, likely through increased glutamate release^34–36^. Behaviorally, autism spectrum disorder mouse models, with hyperactive ventral hippocampus to mPFC excitatory inputs, display impaired social memory that is rescued by inhibition of this circuitry, supporting the notion that overactivation of these neurons may be detrimental to appropriate input–output relationships and their impact on behavior^37^.

Our *ex vivo* data showing that pyramidal neurons are generally quiescent agrees with in vivo studies demonstrating that in the plPFC, glutamatergic neurons are generally quiescent at rest and activity is greatly increased after exposure to an environmental stimulus^38^. In line with these changes in activity, previous work shows that the expression of the immediate early genes *Fos*, *Egr1*, and *Arc* increases at the onset of the active (dark) period^29^. Future work is needed to determine how diurnal changes impact the basal activity of these neurons in vivo and if K^+^ mediated gating mechanisms prevent overactivation of these PFC circuits.

To understand the functional relevance of cell endogenous changes in resting state, we investigated how time-of-day affects action potential dynamics, since action potential firing is a functional measure for information throughput. Notably, this measure changes with time-of-day in the hippocampus, and in response to sleep deprivation in the PFC^39,40^. Action potentials are dependent on voltage-gated ion channels, and changes in K^+^ channel activity can alter action potential firing threshold and kinetics. Consistent with our interpretation that, in male mice, there is a stronger gating mechanism to filter incoming signals during the active period, we discovered that the threshold for action potential firing was increased during the active period. These data suggest that layer 2/3 plPFC pyramidal neurons are not only more hyperpolarized during the light period, but are functionally more difficult to activate, requiring much larger depolarizations to elicit action potentials and relay information downstream. Though somewhat speculative, this could affect a wide range of behaviors, including emotionality, a notion supported by work demonstrating that pharmacological activation of plPFC neurons induces anxiogenic activity in mice, and acute stress enhances glutamatergic transmission in the PFC^13,34,36^.

Neurophysiological sex differences in the PFC, and their respective behavioral outputs, have been documented and partly attributed to differences in synaptic signaling^41,42^. While exploring the effects of time-of-day on these fundamental properties of PFC cells, we fully embraced inclusion of both males and females, since use of both sexes (particularly inclusion of females) improves our overall understanding of brain function^43^. While not designed explicitly as a sex-differences study, our results demonstrate that time-of-day did not strongly influence the basal properties or resting state of PFC pyramidal neurons in female mice. This is consistent with the body of literature demonstrating sex differences in PFC synaptic function and how the PFC responds to environmental challenges. At the synaptic level, there are reported sex differences in glutamate receptor expression and basal release of glutamate throughout the infra- and prelimbic PFC^44–46^. However, not all studies agree, with results on synaptic signaling dependent on animal models and the cell, layer, and regional specificity, which highlights the need for discerning spatial, temporal, and phenotypic differences in neural function. At the circuit and behavioral level, environmental stressors such as social isolation greatly increase aggressive behavior in males, which is mediated by increased synaptic activity from the PFC to basal lateral amygdala (BLA)^47^. Females do not display these same circuit or behavioral changes, and in combination with our findings, it is possible that females have increased resiliency in how the PFC responds to perturbation in the environment. Whether time-of-day effects in these other studies might also contribute to inter-study variability remains unclear, but is another example of why it is important to understand how time-of-day in entrained LD conditions can modulate circuit function.

There are also PFC mediated sex differences in response to environmental and pharmacological stressors^48^. When compared to male rats, female rats can display a lower threshold for impaired working memory after PFC injections of benzodiazepine inverse agonists that activate the stress system^49^. Further, there are clear sex differences in mPFC dendritic growth, microglia activity, and astrocyte morphology in response to stress^50^. We speculate that if the underlying mechanisms that mediate neural function and plasticity are fundamentally different in males and females, then time-of-day changes in neural excitability may not be as crucial to optimal plPFC function in female mice.

Our findings support a cell endogenous mechanism underlying time-of-day changes in the physiology of layer 2/3 plPFC pyramidal neurons. This prompted us to explore how time-of-day impacts intrinsic postsynaptic properties such as ionic currents and overall conductance. There was a large effect on the current density and conductance of pyramidal neurons in male mice. Specifically, when these neurons were hyperpolarized below the equilibrium potential for K^+^, we discovered that current density increased throughout the light period, peaking between the late light period and early dark period. This effect translated into an overall increase in conductance. Given that conductance was highest around the beginning of the active period, when these neurons are most hyperpolarized, we posited that this was due to an increased number of open K^+^ channels and the outflow of K^+^ cations. Consistent with this prediction, when we blocked K^+^ channel mediated currents (by replacing K^+^ with Cs^+^ in our internal recording solution), the time-of-day effect on current density and conductance was completely abolished at voltages near or below the K^+^ equilibrium potential. Although internal Cs^+^ was not sufficient to block the time-of-day effect on current density at depolarized voltage greater than the K^+^ equilibrium potential, it greatly reduced overall current density and conductance.

Circadian desynchronization has myriad consequences for whole-animal physiology. This ranges from peripheral effects on metabolic and immune responses, to cognitive deficits and altered sleep^1,51,52^. Our own prior work showed ECD reduced apical dendrite complexity and impaired cognitive flexibility in male mice. Our present results demonstrate that the consequences of environmental circadian desynchronization extend to the cell function level, reducing information throughput of plPFC neurons by increasing the threshold for action potential firing, which is critical for communication with other brain regions, such as the hippocampus and amygdala.

One caveat of this work is to emphasize it was conducted with mice housed in LD cycles, and as such cannot claim to investigate endogenous circadian contributions (i.e. non-LD cycle) to the observed impact on PFC function. Future work will need to investigate if these rhythms persist in constant darkness, and if they might be controlled by components of the molecular circadian clock or even the SCN. That said, given that nearly all studies focusing on PFC function are conducted in LD cycles, and indeed that the vast majority of non-human animals and humans do not live in constant darkness, we believe our study is therefore better reflective of normative conditions.

It is now well accepted that the mPFC is heterogeneous at the anatomical and physiological levels^15^, and perhaps the temporal dimension needs to be included in this framework. We believe our work reveals that fundamental aspects of cell and circuit function can be impacted by time-of-day and underlines the importance of understanding how daily changes influence neural function throughout the brain. Our findings underscore that if we are to build comprehensive models of cell-circuit-behavioral outputs, we must address the relevant experimental and biological variables that impact these circuits, including biological time.

## Methods

### Mouse model(s)

All animal procedures and experiments were approved by the University of Massachusetts Amherst Institutional Care and Use Committee in accordance with the U.S. Public Health Service Policy on Humane Care and Use of Laboratory Animals and the National Institutes of Health *Guide for the Care and Use of Laboratory Animals* and are in compliance with ARRIVE guidelines^53^. Male and female wild-type mice (Charles River, Wilmington, MA, USA) on a C57BL/6N background (10–16 weeks old) were used for these studies. All mice were group-housed in light-tight housing boxes at 25 °C, under a 12:12-h light:dark (LD) cycle, with food and water available ad libitum. For circadian desynchronization experiments, mice were transferred to a 10:10 h LD cycle and acclimated for at least three weeks prior to experimentation. LD cycles in housing boxes were offset so that experiments from each zeitgeist time (ZT) bin occurred at the same external (real world) time each day. For electrophysiology studies mice were anesthetized in a chamber with isoflurane before euthanasia by decapitation.

### Brain slice electrophysiology

Two mice were simultaneously euthanized 1-h prior to their ZT bin (i.e., mice were euthanized at ZT23 for recording bin ZT0–4). For collections occurring during the dark period, mice were anesthetized under dim red light and quickly transferred to a dissection tray for euthanasia which occurred in less than 60 s. After euthanasia, brains were immediately removed and the forebrain was blocked while bathing in a 0–4 °C oxygenated N-methyl-D-glucamine (NMDG)—4-(2-hydroxyethyl)-1-piperazineethanesulfonic acid (HEPES) cutting solution composed of (mM): 92 NMDG, 2.5 KCl, 1.25 NaH_2_PO_4_, 30 NaHCO_3_, 3 sodium pyruvate, 2 thiourea, 20 HEPES, 10 MgSO_4_, 0.5 CaCl_2_, 25 glucose, 20 sucrose. Cutting solution was brought to pH 7.4 with ~ 17 mL of 5 M HCl^54^. The forebrains were mounted adjacent to each other and sectioned simultaneously on a vibratome (VT1200S, Leica Biosciences, Buffalo Grove, IL, USA) with a sapphire knife (Delaware Diamond Knives, Wilmington, DE, USA) yielding roughly three slices containing the PFC from each (250-μm) per mouse. Slices were transferred and allowed to recover for 30–45 min in room temperature recording artificial cerebrospinal fluid (aCSF) solution composed of (mM): 124 NaCl, 3.7 KCl, 2.6 NaH_2_PO_4_, 26 NaHCO_3_, 2 CaCl_2_, 2 MgSO_4_, 10 glucose. aCSF had a final pH of 7.3–7.4, osmolarity of 307–310 mOsmos, and was continuously bubbled using 95% 0_2_/5% C0_2_. For recordings, brain slices were transferred to a perfusion chamber containing aCSF maintained at 34–37 °C with a flow rate of 1 mL/min. Neurons were visualized using an upright microscope (Olympus BX51W, Tokyo, Japan). Recording electrodes were back-filled with experiment-specific internal solutions as follows (mM): Current-clamp and voltage-clamp; 125 K-gluconate, 10 KCl, 10 NaCl, 5 HEPES, 10 EGTA, 1 MgCl_2_, 3 NaATP and 0.25 NaGTP (liquid-junction potential (LJP) =  ~ 14.5 mV; Predicted E_K_ =  ~ -95 mV). Voltage-clamp cesium-based internal solution; 140 CsCl, 5 MgCl_2_, 1 EGTA, 10 HEPES, 3 NaATP, and 0.25 NaGTP (LJP =  ~ 4.2 mV). All internal solutions were brought to pH 7.3 using KOH or CsOH at 301–304 mOsm. Patch electrodes with a resistance of 3-5MΩ were guided to neurons with an MPC-200-ROE controller and MP285 mechanical manipulator (Sutter Instruments, Novato, CA, USA). Patch-clamp recordings were collected through a UPC-10 USB dual digital amplifier and Patchmaster NEXT recording software (HEKA Elektronik GmbH, Reutlingen, Germany). Current clamp voltage-step protocols were performed from the cell endogenous resting membrane potential and used 500 ms 10-pA steps from − 100 to + 190 pA. Voltage clamp current-step protocols were performed from V_H_ = − 70 mV, and used 10-mV steps from − 120 to + 30 mV. All compounds were obtained from Tocris Cookson, Cayman Chemical, and Sigma Aldrich.

Individual recording locations were plotted (with neurons outside of the target area excluded from analysis) to qualitatively confirm an equal distribution of recording sites between ZT bins 0–4, 6–10, 12–16, and 18–22 (Supplemental Information, Fig. S1 A–D). The electrophysiological heterogeneity of PFC pyramidal neurons is well described. A small percentage (~ 20%) of all recorded neurons had unique characteristics in resting membrane properties and action potential dynamics that were independent of time-of-day (hereafter Type II neurons; Supplemental Information, Fig. S2 A–H). Here we define Type I neurons as non-burst firing pyramidal neurons that are quiescent at rest, have a resting membrane potential (RMP) < -65 mV, membrane resistance < 200 MΩ, antipeak amplitude > -10 mV, and a qualitatively low velocity (measured as ΔmV/ms). Most notably, compared to Type I (most abundant) neurons, Type II neurons (less abundant) displayed a much higher action potential velocity and afterhyperpolarization (Supplemental Information, Fig. S2 A,B). They also had a more depolarized RMP and decreased action potential firing threshold (Supplemental Information, Fig. S2 E,F). These categorical Type II neurons are likely a combination of off-target non-pyramidal neurons, and a small population of phenotypical distinct pyramidal neurons. Due to these clear qualitative and quantitative differences independent of ZT bin, and that they represented a small proportion of recorded neurons, we excluded the far less abundant Type II neurons from analysis in our experiments.

### Experimental design and statistical analysis

Only neurons with input resistance > 70 MΩ were studied. Neurons were not considered for further analysis if series resistance exceeded 50MΩ or drifted > 10% during baseline. Rheobase was calculated as the first current step to elicit an action potential and action potential dynamics (threshold, decay tau, and half-width) were obtained from the first evoked action potential to avoid variance in ion channel function due to repeated action potential firing. G*Power 3.0 software (Franz Faul, Uni Kiel, Germany) was used to conduct our power analysis, for a *p* value of < 0.05 with 90% power. Adequate sample sizes were based upon expected effect sizes from similar experiments. Raw data files were analyzed in the Patchmaster NEXT software or converted using ABF Utility (Synaptosoft) for analysis in MiniAnalysis (Synaptosoft). N-values for analysis and presented in figures represent individual cells. Where applicable, extreme outliers were identified using the ROUT method with a conservative Q = 0.5%. To control for biological variability between groups N = 4–8 mice per group was used. To control for within animal variability 2–3 brain slices were collected per mouse. For experiments including the use of drug, only one cell per slice was used. Statistical comparison of effects between each time-period was made using a full model two-way ANOVA (column, row, and interaction effects) unless otherwise noted. Statistics were calculated using Prism 9 (Graphpad Software, San Diego, CA, USA).

## Supplementary Information

Supplementary Information 1.

Supplementary Information 2.

Supplementary Information 3.

Supplementary Information 4.

Supplementary Information 5.

Supplementary Information 6.

Supplementary Information 7.

Supplementary Information 8.

## Data Availability

The data that support the findings of this study are available from the corresponding author upon reasonable request. Statistical analyses and source data for each figure are included as supplemental information with this manuscript.

## References

[1] Karatsoreos IN, Bhagat S, Bloss EB, Morrison JH, McEwen BS (2011). Disruption of circadian clocks has ramifications for metabolism, brain, and behavior. PNAS.

[2] Woodruff, E. R. *et al.* Coordination between prefrontal cortex clock gene expression and corticosterone contributes to enhanced conditioned fear extinction recall. *eNeuro* **5** (2018). 10.1523/ENEURO.0455-18.2018 PMC6325539 30627637

[3] McCarthy MJ, Welsh DK (2012). Cellular circadian clocks in mood disorders. J. Biol. Rhythms.

[4] Popoli M, Yan Z, McEwen BS, Sanacora G (2012). The stressed synapse: the impact of stress and glucocorticoids on glutamate transmission. Nat. Rev. Neurosci..

[5] Sotres-Bayon F, Cain CK, LeDoux JE (2006). Brain mechanisms of fear extinction: historical perspectives on the contribution of prefrontal cortex. Biol. Psychiat..

[6] Miller EK, Cohen JD (2001). An integrative theory of prefrontal cortex function. Annu. Rev. Neurosci..

[7] Chun LE, Woodruff ER, Morton S, Hinds LR, Spencer RL (2015). Variations in phase and amplitude of rhythmic clock gene expression across prefrontal cortex, hippocampus, amygdala, and hypothalamic paraventricular and suprachiasmatic nuclei of male and female rats. J. Biol. Rhythms.

[8] Woodruff ER, Chun LE, Hinds LR, Spencer RL (2016). Diurnal corticosterone presence and phase modulate clock gene expression in the male rat prefrontal cortex. Endocrinology.

[9] Kawaguchi Y, Kubota Y (1997). GABAergic cell subtypes and their synaptic connections in rat frontal cortex. Cereb Cortex.

[10] Radnikow G, Feldmeyer D (2018). Layer- and cell type-specific modulation of excitatory neuronal activity in the neocortex. Front. Neuroanat..

[11] Vertes RP (2006). Interactions among the medial prefrontal cortex, hippocampus and midline thalamus in emotional and cognitive processing in the rat. Neuroscience.

[12] Saffari R (2016). NPY+-, but not PV+- GABAergic neurons mediated long-range inhibition from infra- to prelimbic cortex. Transl. Psychiatry.

[13] Yuen EY (2009). Acute stress enhances glutamatergic transmission in prefrontal cortex and facilitates working memory. PNAS.

[14] Zaitsev AV, Povysheva NV, Gonzalez-Burgos G, Lewis DA (2012). Electrophysiological classes of layer 2/3 pyramidal cells in monkey prefrontal cortex. J. Neurophysiol..

[15] Moorman DE, James MH, McGlinchey EM, Aston-Jones G (2015). Differential roles of medial prefrontal subregions in the regulation of drug seeking. Brain Res..

[16] Kalmbach BE, Brager DH (2020). Fragile X mental retardation protein modulates somatic D-type K^+^ channels and action potential threshold in the mouse prefrontal cortex. J. Neurophysiol..

[17] Deng W-K, Wang X, Zhou H-C, Luo F (2019). L-type Ca^2+^ channels and charybdotoxin-sensitive Ca^2+^-activated K^+^ channels are required for reduction of GABAergic activity induced by β^2^-adrenoceptor in the prefrontal cortex. Mol. Cell Neurosci..

[18] Workman ER (2015). Rapid antidepressants stimulate the decoupling of GABA(B) receptors from GIRK/Kir3 channels through increased protein stability of 14-3-3η. Mol. Psychiatry.

[19] Bano-Otalora B (2021). Daily electrical activity in the master circadian clock of a diurnal mammal. Elife.

[20] Hablitz LM, Molzof HE, Paul JR, Johnson RL, Gamble KL (2014). Suprachiasmatic nucleus function and circadian entrainment are modulated by G protein-coupled inwardly rectifying (GIRK) channels. J. Physiol..

[21] van Aerde KI, Feldmeyer D (2015). Morphological and physiological characterization of pyramidal neuron subtypes in rat medial prefrontal cortex. Cereb. Cortex.

[22] Piette C (2021). Intracellular properties of deep-layer pyramidal neurons in frontal eye field of macaque monkeys. Front. Synaptic Neurosci..

[23] Chaudhury D, Wang LM, Colwell CS (2005). Circadian regulation of hippocampal long-term potentiation. J. Biol. Rhythms.

[24] Loh DH (2015). Misaligned feeding impairs memories. Elife.

[25] Paul JR (2020). Circadian regulation of membrane physiology in neural oscillators throughout the brain. Eur. J. Neurosci..

[26] Chrobok L (2021). Daily changes in neuronal activities of the dorsal motor nucleus of the vagus under standard and high-fat diet. J. Physiol..

[27] McMartin L, Kiraly M, Heller HC, Madison DV, Ruby NF (2021). Disruption of circadian timing increases synaptic inhibition and reduces cholinergic responsiveness in the dentate gyrus. Hippocampus.

[28] Xu P, Chen A, Li Y, Xing X, Lu H (2019). Medial prefrontal cortex in neurological diseases. Physiol. Genom..

[29] Otsuka T (2020). Adverse effects of circadian disorganization on mood and molecular rhythms in the prefrontal cortex of mice. Neuroscience.

[30] Bechtold DA, Gibbs JE, Loudon AS (2010). Circadian dysfunction in disease. Trends Pharmacol. Sci..

[31] Hou Y (2022). Long-term variable photoperiod exposure impairs the mPFC and induces anxiety and depression-like behavior in male wistar rats. Exp. Neurol..

[32] Harkness JH (2021). Diurnal changes in perineuronal nets and parvalbumin neurons in the rat medial prefrontal cortex. Brain Struct. Funct..

[33] Wallace NK, Pollard F, Savenkova M, Karatsoreos IN (2020). Effect of aging on daily rhythms of lactate metabolism in the medial prefrontal cortex of male mice. Neuroscience.

[34] Li B, Chang L-L, Xi K (2021). Neurotensin 1 receptor in the prelimbic cortex regulates anxiety-like behavior in rats. Prog. Neuropsychopharmacol. Biol. Psychiatry.

[35] Petrie KA (2004). The neurotensin agonist PD149163 increases Fos expression in the prefrontal cortex of the rat. Neuropsychopharmacology.

[36] Saitoh A (2014). Activation of the prelimbic medial prefrontal cortex induces anxiety-like behaviors via N-Methyl-D-aspartate receptor-mediated glutamatergic neurotransmission in mice. J. Neurosci. Res..

[37] Phillips ML, Robinson HA, Pozzo-Miller L (2019). Ventral hippocampal projections to the medial prefrontal cortex regulate social memory. Elife.

[38] Moorman DE, Aston-Jones G (2015). Prefrontal neurons encode context-based response execution and inhibition in reward seeking and extinction. PNAS.

[39] Fusilier AR (2021). Dysregulated clock gene expression and abnormal diurnal regulation of hippocampal inhibitory transmission and spatial memory in amyloid precursor protein transgenic mice. Neurobiol. Dis..

[40] Yan J (2011). Short-term sleep deprivation increases intrinsic excitability of prefrontal cortical neurons. Brain Res..

[41] Andrade S (2012). Effect of progesterone on the expression of GABA(A) receptor subunits in the prefrontal cortex of rats: Implications of sex differences and brain hemisphere. Cell Biochem. Funct..

[42] de Velasco EMF (2015). Sex differences in GABABR-GIRK signaling in layer 5/6 pyramidal neurons of the mouse prelimbic cortex. Neuropharmacology.

[43] Shansky RM, Murphy AZ (2021). Considering sex as a biological variable will require a global shift in science culture. Nat. Neurosci..

[44] Perry CJ, Campbell EJ, Drummond KD, Lum JS, Kim JH (2021). Sex differences in the neurochemistry of frontal cortex: Impact of early life stress. J. Neurochem..

[45] Pena-Bravo JI, Penrod R, Reichel CM, Lavin A (2019). Methamphetamine self-administration elicits sex-related changes in postsynaptic glutamate transmission in the prefrontal cortex. eNeuro.

[46] Knouse MC (2022). Sex differences in the medial prefrontal cortical glutamate system. Biol. Sex Differ..

[47] Wang Z-J (2022). Molecular and cellular mechanisms for differential effects of chronic social isolation stress in males and females. Mol. Psychiatry.

[48] Yuen EY, Wei J, Yan Z (2016). Estrogen in prefrontal cortex blocks stress-induced cognitive impairments in female rats. J. Steroid Biochem. Mol. Biol..

[49] Shansky RM (2004). Estrogen mediates sex differences in stress-induced prefrontal cortex dysfunction. Mol. Psychiatry.

[50] Bollinger JL, Salinas I, Fender E, Sengelaub DR, Wellman CL (2019). Gonadal hormones differentially regulate sex-specific stress effects on glia in the medial prefrontal cortex. J. Neuroendocrinol..

[51] Pearson GL, Savenkova M, Barnwell JJ, Karatsoreos IN (2020). Circadian desynchronization alters metabolic and immune responses following lipopolysaccharide inoculation in male mice. Brain Behav. Immun..

[52] Phillips DJ, Savenkova MI, Karatsoreos IN (2015). Environmental disruption of the circadian clock leads to altered sleep and immune responses in mouse. Brain Behav. Immun..

[53] du Sert NP (2020). The ARRIVE guidelines 2.0: Updated guidelines for reporting animal research. PLOS Biol..

[54] Ting JT (2018). Preparation of acute brain slices using an optimized N-methyl-D-glucamine protective recovery method. J. Vis. Exp. JoVE.

