# Supplementary material for: Circadian desynchronization disrupts physiological rhythms of prefrontal cortex pyramidal neurons in mice
Source: Sci Rep. 2023 Jun 6;13:9181. doi: 10.1038/s41598-023-35898-8 (PMC10244337; doi:10.1038/s41598-023-35898-8)
Supplement: Supplementary file 7 — Supplementary Information 7. [file 41598_2023_35898_MOESM7_ESM.docx]

**Supplemental Information for:**

**Circadian desynchronization disrupts physiological rhythms of prefrontal cortex pyramidal neurons in mice**

Brandon L. Roberts^1^ and Ilia N. Karatsoreos^1^*

^1^ Neuroscience and Behavior Program, and Department of Psychological and Brain Sciences, University of Massachusetts Amherst, Amherst, MA 01003, USA

***Correspondence should be sent to:**

Ilia N. Karatsoreos, Ph.D.

Department of Psychological and Brain Sciences

University of Massachusetts Amherst

Tobin Hall, 135 Hicks Way

Amherst, MA 01003

**Email:**  [ikaratsoreos@umass.edu](mailto:ikaratsoreos@umass.edu)

**This file includes:**

Figures S1 to S2

1. **Supporting Figures**


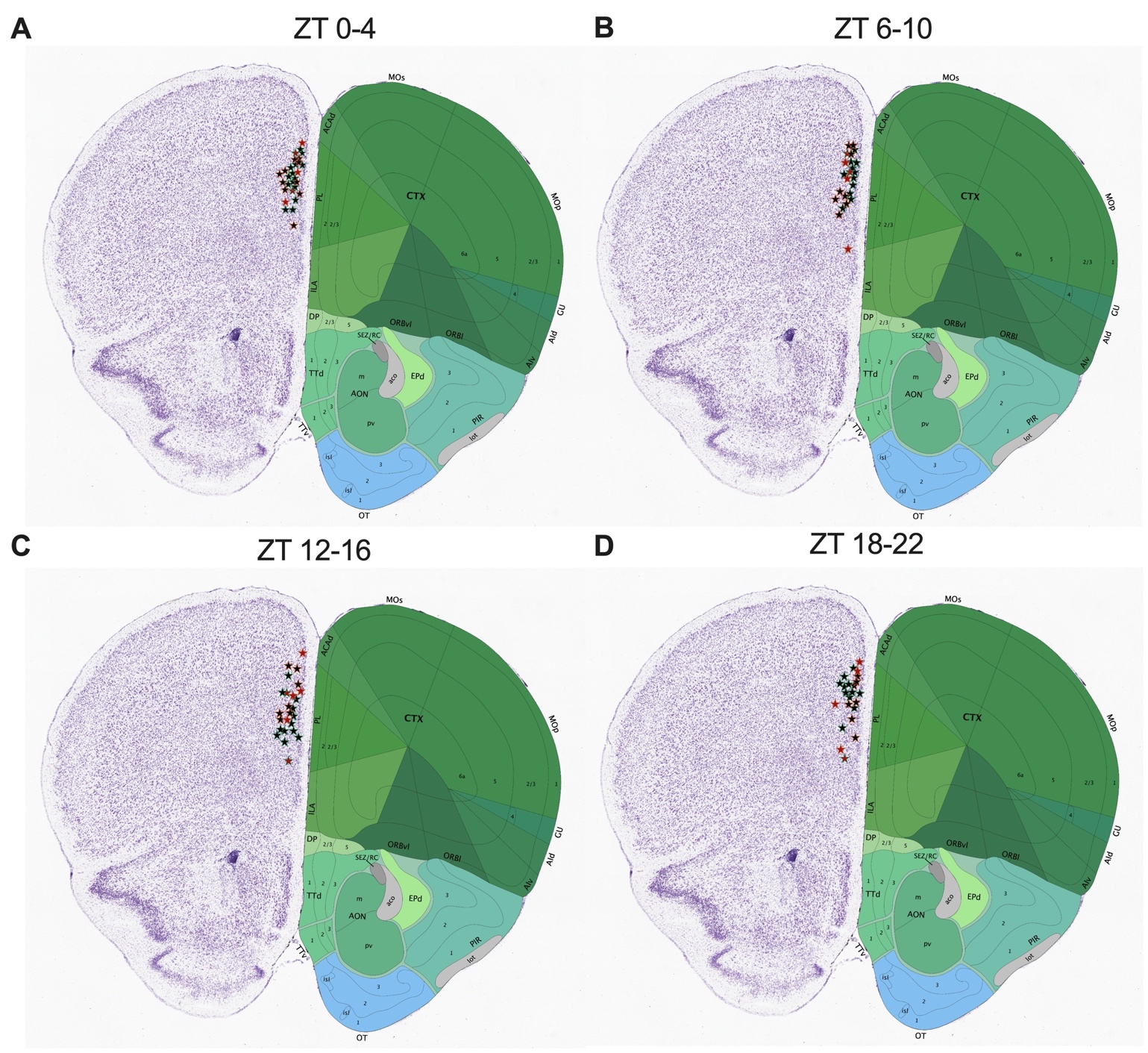


**Fig. S1.** *Recording map for layer 2/3 plPFC pyramidal neurons.* Coronal sections of forebrain showing individual recording sites from majority of neurons that were imaged at **(A)** ZT0-4, **(B)** 6-10, **(C)** 12-16 and **(D)** 18-22 for basal membrane property, sEPSC, and evoked action potential experiments in male (*bluish green outline*) and female (*vermillian outline*) mice. Stars filled with black represent ‘Type I’ neurons included for analysis and red stars represent Type II/III neurons excluded from analysis.

**Fig. S2.** *Categories and distinct physiological characteristics of plPFC neurons.* **(A)** Representative evoked action potential traces and phase plot diagram of first five action potentials (*bottom*) illustrating differences in velocity, trajectory, and amplitude in Type I and **(B)** Type II neurons. **(C)** Boxplot comparison of membrane resistance (Rm; t = 6.656, df = 131; *p* <0.001), **(D)** membrane capacitance (Cm; t = 2.003, df = 125; *p* = 0.05), **(E)** resting membrane potential (RMP t = 8.133, df = 126; *p* <0.001), **(F)** action potential (AP) threshold (t = 2.674, df = 113; *p* = 0.009), **(G)** antipeak amplitude (t = 8.27, df = 114; *p* < 0.001), and **(H)** Percentage of recorded cells displaying Type I or Type II characteristics (combined among all ZT bins and sexes; calculated by *n* values from AP threshold). Unpaired student t-test, * *p< 0.05*, ** *p< 0.01*, ****p< 0.001*.
